# Oncogenic driver mutations in Swiss never smoker patients with lung adenocarcinoma and correlation with clinicopathologic characteristics and outcome

**DOI:** 10.1371/journal.pone.0220691

**Published:** 2019-08-06

**Authors:** Claudia Grosse, Alex Soltermann, Markus Rechsteiner, Alexandra Grosse

**Affiliations:** 1 Institute of Pathology and Molecular Pathology, Clinical Pathology, University Hospital Zurich, Zurich, Switzerland; 2 Institute of Pathology and Molecular Pathology, Diagnostic Molecular Pathology, University Hospital Zurich, Zurich, Switzerland; University of Toronto, CANADA

## Abstract

**Purpose:**

Lung cancer in never smokers is recognized as a distinct molecular, clinicopathologic and epidemiologic entity. The aim of the study was to investigate the molecular profile in Swiss never smokers with lung adenocarcinoma and to correlate the mutation status with clinicopathologic and demographic patient characteristics and outcome.

**Methods:**

One hundred thirty-eight never smokers diagnosed with lung adenocarcinoma at the University Hospital Zurich between 2011–2018 were included in the study. Data from the electronic medical records were reviewed to characterize clinicopathologic and demographic features, molecular profile, treatment and outcome.

**Results:**

The majority of patients were female (58.7%) with a median age at diagnosis of 64.5 years (range, 27.1–94.2 years). The most common mutations were *EGFR* (58.7%) followed by *ALK* (12.3%), *TP53* (5.8%), *MET* (5.8%), *KRAS* (4.3%), *ERBB2* (4.3%), *PIK3CA* (2.9%), *BRAF* (2.2%), *ROS1* (1.4%), *RET* (1.4%), CTNNB1 (0.7%), PARP1 (0.7%), TET1 (0.7%) and PIK3CG (0.7%). Median overall survival (mOS) was 51.0 months (mo). Early clinical stage (*p* = 0.002) and treatment with targeted therapy (HR 2.53, 95% CI 1.35–4.74, *p* = 0.004) were independently associated with longer mOS. Patients with oncogenic driver mutations had significantly longer mOS (52.2 mo) compared to patients without mutations (16.9 mo) (HR 3.38, 95% CI 1.52–7.55, *p* = 0.003). Besides, patients with *EGFR* mutated (57.8 mo) or *ALK* rearranged (59.9 mo) tumors had significantly longer mOS compared to the *EGFR* wildtype (35.0 mo), *ALK* wildtype (46.5 mo) and pan-negative (16.9 mo) cohorts (HR 2.35, 95% CI 1.37–4.04, *p* = 0.002; HR 7.80, 95% CI 3.28–18.55, *p* < 0.001; HR 3.96, 95% CI 1.21–12.95, *p* = 0.023 and HR 34.78, 95% CI 3.48–34.65, *p* = 0.003).

**Conclusion:**

Never smokers with lung adenocarcinoma display distinct clinicopathologic and molecular features and are characterized by a high incidence of targetable mutations. Never smokers with targetable mutations have significantly longer survival compared to patients without mutations.

## Introduction

Although the majority of lung cancer cases are associated with tobacco smoking, a considerable proportion (~10–40%) of patients develop lung cancer without a significant personal history of tobacco use [1–5]. Lung cancer in never smokers (LCINS) constitutes an entity with distinct gender, geographic, clinicopathologic and molecular characteristics compared to lung cancer occurring in smokers [2,3]. The majority (~65%-87% [1,6–8]) of never smoker patients with lung cancer are women, and the incidence of LCINS is significantly higher in certain geographic regions, including East Asia, than in the United States and in Europe [1,9,10]. Molecular profiling studies have shown that never smokers with lung adenocarcinoma harbor significantly lower somatic mutation burden than smokers with the same disease [11]. Besides, C > A transversions are more common in smokers, while C > T transitions occur more frequently in never smokers [12]. *EGFR* mutations and *ALK* rearrangements appear to be more frequent in never smokers than in smokers, whereas *KRAS* and *BRAF* mutations are less common in this subset of patients [6,13,14]. The majority of previous studies that have characterized the genomic alterations of LCINS have come from East Asia due to the high incidence of LCINS in this geographic region. In contrast, data on the molecular characteristics of LCINS in Western populations are scarce [1,6].

The aim of the current study was to analyze the molecular features of Swiss never smoker patients with lung adenocarcinoma and to correlate the mutation status with demographic and clinicopathologic characteristics and outcome. Because lung adenocarcinoma is the most common histologic subtype in never smokers with non-small cell lung cancer (NSCLC) accounting for up to 94% of LCINS [7], we focused on never smokers with adenocarcinoma histology.

## Methods

### Patients and data collection

We performed a retrospective analysis of demographic, clinical and pathologic data stored in the electronic medical record system at the University Hospital Zurich of all never smokers with a pathologic diagnosis of stage I-IV lung adenocarcinoma, radio-chemotherapy and targeted therapy naïve, diagnosed at our institution between January 2011 and January 2018. Inclusion criteria were: 1) being a self-declared never smoker; 2) a diagnosis of histologically and/or cytologically confirmed lung adenocarcinoma; 3) radio-chemotherapy and targeted therapy naïve; and 4) tissue blocks/cell blocks with adequate tumor cellularity. Exclusion criteria were: 1) non-adenocarcinoma histology; 2) previous chemotherapy, targeted therapy or radiotherapy; and 3) insufficient tumor material. Never smokers were defined as individuals who had smoked less than 100 cigarettes in their lifetime. The following data were retrieved from the electronic patient record system: gender, ethnicity, age at diagnosis, clinical stage at diagnosis, TNM stage at diagnosis (according to the Union for International Cancer Control (UICC) TNM classification of malignant tumors, 8^th^ edition [15]), tumor location, tumor size, type and duration of systemic treatment, and survival history. Overall survival was measured from the date of pathologic diagnosis until the date of death. Patients were censored on May 31, 2018 if they were alive. Patients without a known date of death were censored at the time of last follow-up. The diagnosis of lung adenocarcinoma was made on hematoxylin and eosin stained and immunostained formalin-fixed, paraffin-embedded tissue sections from resection or biopsy specimens and/or on cytologic samples based on the 2015 World Health Organization classification for lung tumors [16]. The study was approved by the Cantonal Ethics Committee of Zurich (StV-No. 2018–01919), and written informed consent was obtained from all patients, including written permission of two patients to publish their PET-CT scans.

### Molecular analysis and immunohistochemistry

Tumor tissues from core biopsies (58.7%), resection specimens (30.4%) and cytologic samples (10.9%) were used to perform all molecular analyses according to the National Comprehensive Cancer Network (NCCN) and Swiss Society of Pathology (SSPath) guidelines, as previously described [17]. Specific genotyping methods utilized included Sanger sequencing (SS) of *EGFR*, *KRAS*, *ERBB2* and *BRAF*, immunohistochemistry (IHC)/immunocytochemistry (ICC) assays for *ALK* and *ROS1*, break-apart fluorescence in situ hybridization (FISH) for *ALK*, *ROS1* and *RET* and targeted DNA- and RNA-based next-generation sequencing (NGS). Targeted DNA- and RNA-based NGS was performed in 69 patients using different customer panels during the study period, including the Ion AmpliSeq Colon and Lung Cancer panel 2 (CLP2), Ion AmpliSeq Fusion Lung Cancer Research panel (LFP), and Oncomine DNA panel for Solid Tumors and Fusion Transcripts (Thermo Fisher Scientific/Life Technologies, Carlsbad, California, USA). We used the Ion Library Quantitation kit (Thermo Fisher Scientific) for quantification of DNA and RNA libraries, the Ion One Touch 200 Template Kit v2 DL (lately replaced by the Ion Hi-Q Chef Kit and the Ion Chef System) (Thermo Fisher Scientific) for emulsion polymerase chain reaction (PCR) and template preparation, and the Ion Personal Genome Machine 200 Kit v2 (lately replaced by the Ion Personal Genome Machine Hi-Q Sequencing Kit) (Thermo Fisher Scientific) as sequencing platform. For Sanger sequencing, we used the Illustra GFX PCR DNA and Gel Band Purification Kit (GE Healthcare Life Sciences, Buckinghamshire, UK) for purification of amplified DNA fragments, the Genetic Analyzer 3130x1 (Applied Biosystems, Foster City, CA, USA) for sequencing and the Sequencher 5.1 (Gene Code Corporation, Ann Arbor, MI, USA) for data analysis. *ALK* and *ROS1* immunohistochemistry (IHC)/immunocytochemistry (ICC) was performed on the automated immunostainer DiscoveryUltra (Roche Ventana) using a mouse anti-human ALK monoclonal antibody (clone 5A4, Leica Biosystems) and a rabbit anti-human ROS1 monoclonal antibody (clone D4D6, Cell Signaling Technology). *ALK* or *ROS1* IHC/ICC positive cases were confirmed by FISH using the Vysis LSI ALK Dual Color Break Apart Rearrangement Probe (Abbott Molecular, Baar, Switzerland) and the Zyto*Light* SPEC ROS1 Dual Color Break Apart Probe (Zytovision GmbH, Bremerhaven, Germany). FISH testing for *RET* rearrangement was performed using the Zyto*Light* SPEC RET Dual Break Apart Probe (Zytovision GmbH, Bremerhaven, Germany).

### Statistical analysis

Descriptive statistics were applied to describe the patient characteristics of the study cohort. The results are presented as frequencies and percentages for categorical variables and as mean ± standard deviation, median and range for continuous variables. Univariate analysis was performed to assess associations between mutation status and clinicopathologic and demographic characteristics, using chi-square test or Fisher exact test for categorical variables and t test or non-parametric Mann-Whitney test for continuous variables. Median overall survival (mOS) was estimated using the Kaplan-Meier method, and survival curves were compared with the log-rank test. Adjusted hazard ratios were calculated using Cox regression models (with a backward stepwise selection method) that included age, gender, clinical stage and treatment as independent variables. *P*-values < 0.05 were considered statistically significant. All statistical analyses were performed using SPSS Statistics software (version 24.0, IBM, Ehningen, Germany).

## Results

### Patients

We identified 138 never smokers who fulfilled the inclusion criteria. Patient and tumor characteristics are summarized in Table 1. Most patients were female (81/138, 58.7%), with a median age at diagnosis of 64.5 years (mean, 63.3 ± 13.1 years; range, 27.1–94.2 years), and presented with clinical stage IV (95/138, 68.8%). 15.2% (21/138) of patients had brain metastases at diagnosis, and 12.3% (17/138) of patients developed brain metastases during follow-up. Of the entire study population, 8 (8/138, 5.8%) patients were lost to follow-up, and 130 (130/138, 94.2%) patients, including 116 patients with oncogenic driver mutations, were followed up for a median time of 28.5 months (mo) (mean, 31.6 ± 19.0 mo, range, 2–84 mo). 56 (56/130, 43.1%) patients died during follow-up, while 74 (74/130, 56.9%) patients were alive at last follow-up, including 45 (45/130, 34.6%) patients with stable disease and 29 (29/130, 22.3%) patients with progressive disease. Median OS for the evaluable patients was 51.0 mo (mean, 52.0 ± 3.2 mo). 46 (46/130, 35.4%) patients received one treatment modality, while 84 (84/130, 64.6%) patients were treated with combined therapies. Treatment consisted of surgery in 54 (54/130, 41.5%) patients, chemotherapy in 88 (88/130, 67.7%) patients, radiotherapy in 40 (40/130, 30.8%) patients and molecular targeted therapy in 61 (61/130, 46.9%) patients. Patients with *EGFR* mutation and *ALK* rearrangement were treated with targeted therapy in 59.8% (55/92). The majority of patients with stage IV disease for whom information about treatment was available (86/89, 96.6%) received first-line systemic (targeted or chemotherapy) treatment. Most of the chemotherapy-treated patients (including those who underwent chemo-radiotherapy) received platinum-based regimens (94%). Among individuals for whom information about treatment was available, 92.9% (26/28) of patients with stage I-IIIA, 89.7% (35/39) of patients with stage I-IIIB and 87.8% (36/41) of patients with stage I-IIIC underwent surgical resection.

**Table 1:** Patient and tumor characteristics.

Clinical and demographic characteristics			
	All patients (*n* = 138)		All patients (*n* = 138)
Age (years)		Distribution	
Median (range)	64.5 (27.1–94.2)	Central	32 (23.2)
Mean	63.3 ± 13.1	Peripheral	83 (60.1)
Gender		Central and peripheral	23 (16.7)
Male	57 (41.3)	T stage	
Female	81 (58.7)	T1	19 (13.8)
Ethnicity		T2	39 (28.3)
Caucasian	130 (94.2)	T3	25 (18.1)
Asian	4 (2.9)	T4	55 (39.9)
Other	4 (2.9)	Lymph node involvement	105 (76.1)
Clinical stage at diagnosis		N stage	
I	9 (6.5)	N0	33 (23.9)
II	11 (8.0)	N1	16 (11.6)
III	23 (16.7)	N2	39 (28.3)
IV	95 (68.8)	N3	50 (36.2)
Localization		Extrathoracic metastasis/-es	65 (47.1)
Right upper lobe	31 (22.5)	M stage	
Right lower lobe	14 (10.1)	M0	43 (31.2)
Middle lobe	8 (5.8)	M1a	30 (21.7)
Left upper lobe	27 (19.6)	M1b	18 (13.0)
Left lower lobe	22 (15.9)	M1c	47 (34.1)
Lingula	2 (1.4)	Brain metastases at diagnosis	21 (15.2)
Involvement of two lobes	34 (24.6)	Brain metastases at diagnosis and during follow-up	38 (27.5)
Size (mm)			
Mean	46.3 ± 24.5	Malignant pleural effusion	40 (29.0)
Molecular characteristics			
	All patients (*n* = 138)		Patients tested†
*EGFR*	81/138 (58.7)	*EGFR*	81/138 (58.7)
*ALK*	17/138 (12.3)	*ALK*	17/108 (15.7)
*MET*	8/138 (5.8)	*MET*	8/53 (15.1)‡
*KRAS*	6/138 (4.3)	*KRAS*	6/114 (5.3)
*ERBB2*	6/138 (4.3)	*ERBB2*	6/74 (8.1)
*PIK3CA*	4/138 (2.9)	*PIK3CA*	4/58 (6.9)
*BRAF*	3/138 (2.2)	*BRAF*	3/72 (4.2)
*ROS1*	2/138 (1.4)	*ROS1*	2/78 (2.6)
*RET*	2/138 (1.4)	*RET*	2/52 (3.8)
Other	10/138 (7.2)	Other	10/56 (17.9)
Triple negative	34/138 (24.6)	Triple negative	34/104 (32.7)
Pan-negative	13/138 (9.4)	Pan-negative	13/56 (23.2)
Treatment			
	Patients with information about treatment (*n* = 130)		
Surgery	54 (41.5)		
Chemotherapy	88 (67.7)		
Radiotherapy	40 (30.8)		
Targeted therapy	61 (46.9)		
*EGFR*	45/76 (59.2)		
*ALK*	10/16 (62.5)		
*ERBB2*	2/6 (33.3)		
*BRAF*	1/3 (33.3)		
*MET*	2/8 (25.0)		
*PIK3CA*	1/1 (100)		

Data are mean values ± standard deviations for continuous variables and number of patients with percentages in parentheses for categorical variables.

^†^Percentages in parentheses refer to the number of tested patients.

^‡^Exclusively patients tested for *MET* mutations (including *MET* exon 14 skipping mutations) with NGS.

### Mutation analyses

At least one mutation was present in 125 (90.6%) of 138 analyzed tumors. *EGFR* (exon 19 deletions– 45/81, 55.6%, exon 21 p.L858R point mutation– 26/81, 32.1%) was the most common mutation (81/138, 58.7%, NGS: 33, SS: 48) (Table 2), followed by *ALK* (17/138, 12.3%, NGS: 3, FISH: 14), *TP53* (8/138, 5.8%, NGS: 8), *MET* (8/138, 5.8%, NGS: 8), *KRAS* (6/138, 4.3%, NGS: 3, SS: 3), *ERBB2* (6/138, 4.3%, NGS: 3, SS: 3), *PIK3CA* (4/138, 2.9%, NGS: 4), *BRAF* (3/138, 2.2%, NGS: 2, SS: 1), *ROS1* (2/138, 1.4%, NGS: 2), *RET* (2/138, 1.4%, NGS: 1, FISH: 1), *CTNNB1* (1/138, 0.7%, NGS: 1), *PARP1* (1/138, 0.7%, NGS: 1), *TET1* (1/138, 0.7%, NGS: 1) and *PIK3CG* (1/138, 0.7%, NGS: 1) (Table 1). Doublet *EGFR* mutations were present in 9 (9/138, 6.5%) tumors, including 4 tumors with p.L858R and non-p.L858R missense mutations and 5 tumors with two non-p.L858R missense mutations (S1 Table). Of 21 tumors with multiple mutations, 17 (17/21, 81.0%) had *EGFR* as a co-mutation (S1 Table). Mutations were not detected in 13 (9.4%) of 138 tumors (pan-negative), and 34 (24.6%) of 138 tumors were triple-negative (*EGFR* negative/*ALK* negative/*KRAS* negative) (Table 1). *KRAS* mutations were most frequently located in exon 2/codon 12 (5/6, 83.3%), and p.G12V (3/6, 50.0%) was the most common subtype (Table 2). *ERBB2* mutations were exclusively exon 20 insertions/duplications, and the most frequent *ERBB2* mutation was p.A771_M774dup (2/6, 33.3%) (Table 2). Of 21 patients with brain metastases at diagnosis, 15 (15/21, 71.4%) had *EGFR* mutation, 2 (2/21, 9.5%) were pan-negative, 2 (2/21, 9.5%) had *ALK* translocation, 1 (1/21, 4.8%) harbored *PIK3CA* mutation and 1 (1/21, 4.8%) harbored *MET* exon 14 skipping mutation.

**Table 2:** Oncogenic driver mutations in never smokers.

EGFR mutation (*n* = 81)				
	cDNA change	Amino acid change	Frequency	Percentage
Exon 21	c.2573T>G	p.L858R	26	32.1
Exon 21	c.2497T>G	p.L833V	1	1.2
Exon 21	c.2560A>G	p.T854A	1	1.2
Exon 21	c.2579A>T	p.K860I	1	1.2
Exon 20	c.2303_2311dup	p.S768_D770dup	2	2.5
Exon 20	c.2303G>T	p.S768I	2	2.5
Exon 20	c.2320G>A	p.V774M	1	1.2
Exon 20	c.2389T>A	p.C797S	1	1.2
Exon 20	c.2320_2321insCACATG	p.H773_V774insAH	1	1.2
Exon 20	c.2310_2311insGGGC	p.D770_N771insG	1	1.2
Exon 20	c.2317_2322	p.H773_V774dup	1	1.2
Exon 19	c.2235_2249del/c.2236_2250del†	p.E746_A750del	26	32.1
Exon 19	c.2240_2257del	p.L747_P753delinsS	5	6.2
Exon 19	c.2237_2255delinsT	p.E746_S752delinsV	3	3.7
Exon 19	c.2254_2277del	p.S752_I759del	2	2.5
Exon 19	c.2240_2254del	p.L747_T751del	2	2.5
Exon 19	c.2239_2256del	p.L747_S752del	2	2.5
Exon 19	c.2239_2252delinsCA	p.L747_T751delinsQ	1	1.2
Exon 19	c.2239_2248delinsC	p.L747_A750delinsP	1	1.2
Exon 19	c.2239_2258delinsCA	p.L747_P753delinsQ	1	1.2
Exon 19	c.2239_2251delinsC	p.L747_T751delinsP	1	1.2
Exon 19	c.2251_2277del	p.T751_I759delinsS	1	1.2
Exon 19	c.2281G>T	p.D761Y	1	1.2
Exon 18	c.2156G>C	p.G719A	2	2.5
Exon 18	c.2126A>C	p.E709A	1	1.2
Exon 18	c.2126A>G	p.E709G	1	1.2
Exon 18	c.2155G>T	p.G719C	1	1.2
Exon 18	c.2142G>C	p.K714N	1	1.2
*KRAS* mutations (*n* = 6)				
	cDNA change	Amino acid change	Frequency	Percentage
Codon 12/Exon 2	c.35G>T	p.G12V	3	50.0
Codon 12/Exon 2	c.34G>T	p.G12C	1	16.7
Codon 12/Exon 2	c.35G>A	p.G12D	1	16.7
Codon 61/Exon 3	c.182A>T	p.Q61L	1	16.7
*BRAF* mutations (*n* = 3)				
	cDNA change	Amino acid change	Frequency	Percentage
Exon 15	c.1799T>A	p.V600E	2	66.7
Exon 15	c.1799T>A	p.V600G	1	33.3
*ERBB2* mutations (*n* = 6)				
	cDNA change	Amino acid change	Frequency	Percentage
Exon 20	c.2324_2325ins	p.E770_A771ins	1	16.7
Exon 20	c.2313_2324dup	p.A771_M774dup	2	33.3
Exon 20	c.2326_2327insTGT	p.G776delinsVC	1	16.7
Exon 20	c.2310_2311ins12	p.E770_A771insAYVM	1	16.7
Exon 20	c.2331_2339dup	p.G778_P780dup	1	16.7
*PIK3CA* (*n* = 4)				
	cDNA change	Amino acid change	Frequency	Percentage
Exon 10	c.1633G>A	p.E545K	2	50.0
Exon 10	c.1624G>A	p.E542K	1	25.0
Exon 21	c.3140A>G	p.H1047R	1	25.0

^†^c.2235_2249del: *n* = 18; c.2236_2250del: *n* = 8.

### Correlation with clinicopathologic characteristics

**Fig 1:**
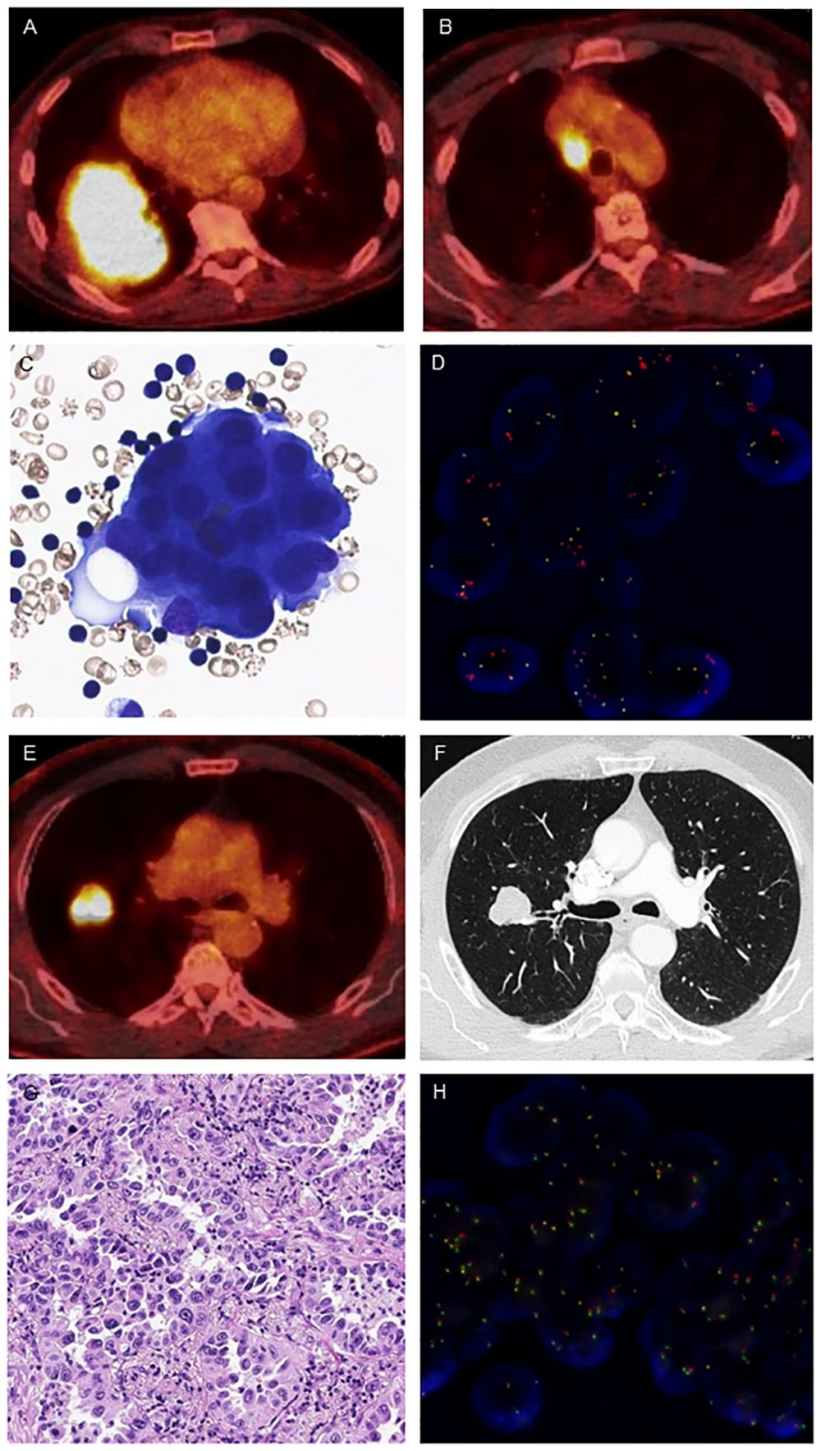
*ALK* rearranged vs. *ALK* wildtype lung adenocarcinoma in never smokers. (A,B) PET-CT scans showing a large tumor mass in the right lower lobe together with ipsilateral mediastinal lymph node metastasis. (C) Adenocarcinoma morphology on cytological sample (May-Grünwald stain, x 400). (D) Positive *ALK* fluorescence in situ hybridization (FISH) with split signals and mainly isolated red signals. (E) PET-CT scan and (F) axial CT image showing a tumor nodule in the right upper lobe without mediastinal lymph node metastases. (G) Adenocarcinoma histology on resection specimen (hematoxylin & eosin stain, x 200). (H) *ALK* FISH negativity with fusion signals.

No statistically significant differences were found between male and female never smokers with respect to clinical stage, TNM stage, presence of brain metastasis at diagnosis and during follow-up, tumor location, mean tumor size, mean patient age at diagnosis and the frequency of oncogenic driver mutations (S2 Table). Comparative analyses of *EGFR* mutated tumors with *EGFR* wildtype, pan-negative and *ALK* positive tumors showed that *EGFR* mutated tumors more frequently had extrathoracic metastases at diagnosis compared to *EGFR* wildtype tumors (55.6% vs. 35.1%, *p* = 0.018) and were more commonly located in the right upper lobe compared to *EGFR* wildtype, pan-negative and *ALK* positive tumors (28.4% vs. 14.0%, *p* = 0.047; 28.4% vs. 0.0%, *p* = 0.033 and 28.4% vs. 0.0%, *p* = 0.010) (Table 3). There was a significant difference in mean age between patients with *EGFR* mutated tumors and those without identifiable mutations (61.9 ± 13.4 vs. 71.9 ± 9.2 years, *p* = 0.011) (Table 3) as well as between patients with mutations in their tumors and the pan-negative cohort (62.4 ± 13.2 vs. 71.9 ± 9.2 years, *p* = 0.012) (S3 Table). *ALK* rearranged lung adenocarcinomas were less frequently located in the right upper lobe (0.0% vs. 25.6%, *p* = 0.013) compared to *ALK* wildtype tumors and more commonly showed ipsilateral mediastinal or subcarinal lymph node metastasis (N2) compared to *EGFR* mutated and *ALK* wildtype tumors (52.9% vs. 22.2%, *p* = 0.016 and 52.9% vs. 24.8%, *p* = 0.022) (Tables 3 and 4) (Fig 1). Comparative analysis of patients with tumors harboring exon 21 p.L858R point mutation and patients with tumors carrying exon 19 deletions showed significant differences in mean age, tumor location (central vs. peripheral) and the frequency of ipsilateral mediastinal or subcarinal lymph node metastasis (N2) and malignant pleural effusion (S4 Table). Patients with brain metastases at diagnosis more frequently had T4 tumors and contralateral mediastinal, hilar or supraclavicular lymph node metastasis (N3) compared to patients without brain metastases at diagnosis (61.9% vs. 35.9%, *p* = 0.025 and 61.9% vs. 31.6%, *p* = 0.008) (S5 Table). Likewise, patients with brain metastases at diagnosis and during follow-up more commonly showed lymph node metastases compared to patients without brain metastases (92.1% vs. 70.0%, *p* = 0.007) (S6 Table). Lung adenocarcinoma diagnosed in patients < 45 years was more commonly associated with lymph node metastasis (100.0% vs. 73.4%, *p* = 0.022), notably contralateral mediastinal, hilar or supraclavicular lymph node metastasis (N3) (64.3% vs. 33.1%, *p* = 0.021), and extrathoracic metastases (78.6% vs. 43.5%, *p* = 0.013) compared to tumors diagnosed in patients > 45 years (S7 Table).

**Table 3:** Comparison of *EGFR* mutated tumors with *EGFR* wildtype, pan-negative and *ALK* positive tumors.

Variable	*EGFR* mt (*n* = 81)	*EGFR* wt (*n* = 57)	*p*	Pan-negative (*n* = 13)	*p*	*ALK* positive (*n* = 17)	*p*
Age (years)	61.9 ± 13.4	65.3 ± 12.6	0.133	71.9 ± 9.2	**0.011**	64.2 ± 14.0	0.515
Gender			0.225		0.250		0.097
Male	30 (37.0)	27 (47.4)		7 (53.8)		10 (58.8)	
Female	51 (63.0)	30 (52.6)		6 (46.2)		7 (41.2)	
Clinical stage							
I	6 (7.4)	3 (5.3)	0.736	1 (7.7)	0.971	0 (0.0)	0.586
II	5 (6.2)	6 (10.5)	0.361	1 (7.7)	0.839	2 (11.8)	0.601
III	10 (12.3)	13 (22.8)	0.104	3 (23.1)	0.381	5 (29.4)	0.130
IV	60 (74.1)	35 (61.4)	0.114	8 (61.5)	0.339	10 (58.8)	0.242
T stage							
T1	11 (13.6)	8 (14.0)	0.939	0 (0.0)	0.352	3 (17.6)	0.705
T2	25 (30.9)	14 (24.6)	0.418	4 (30.8)	0.995	4 (23.5)	0.547
T3	18 (22.2)	7 (12.3)	0.135	1 (7.7)	0.455	2 (11.8)	0.670
T4	27 (33.3)	28 (49.1)	0.062	8 (61.5)	0.066	2 (11.8)	0.537
LN meta	59 (72.8)	46 (80.7)	0.286	10 (76.9)	0.754	15 (88.2)	0.228
N stage							
N0	22 (27.2)	11 (19.3)	0.286	3 (23.1)	0.754	2 (11.8)	0.228
N1	10 (12.3)	6 (10.5)	0.742	2 (15.4)	0.670	1 (5.9)	0.683
N2	18 (22.2)	21 (36.8)	0.060	3 (23.1)	0.945	9 (52.9)	**0.016**
N3	31 (38.3)	19 (33.3)	0.552	5 (38.5)	0.990	5 (29.4)	0.491
Extrathoracic meta	45 (55.6)	20 (35.1)	**0.018**	5 (38.5)	0.252	5 (29.4)	0.050
M stage							
M0	21 (25.9)	22 (38.6)	0.114	5 (38.5)	0.339	7 (41.2)	0.242
M1a	15 (18.5)	15 (26.3)	0.274	5 (38.5)	0.709	5 (29.4)	0.330
M1b	12 (14.8)	6 (10.5)	0.461	1 (7.7)	0.686	0 (0.0)	0.119
M1c	33 (40.7)	14 (24.6)	**0.048**	4 (30.8)	0.495	5 (29.4)	0.383
Localization							
Right upper lobe	23 (28.4)	8 (14.0)	**0.047**	0 (0.0)	**0.033**	0 (0.0)	**0.010**
Right lower lobe	6 (7.4)	8 (14.0)	0.204	3 (23.1)	0.107	2 (11.8)	0.624
Middle lobe	5 (6.2)	3 (5.3)	0.821	1 (7.7)	0.839	2 (11.8)	0.601
Left upper lobe	15 (18.5)	12 (21.1)	0.712	3 (23.1)	0.709	4 (23.5)	0.736
Left lower lobe	12 (14.8)	10 (17.5)	0.666	2 (15.4)	0.957	3 (17.6)	0.721
Lingula	0 (0.0)	2 (3.5)	0.169	0 (0.0)	-	1 (5.9)	0.173
Mixed	20 (24.7)	14 (24.6)	0.986	4 (30.8)	0.733	5 (29.4)	0.761
Distribution							
Central	18 (22,2)	14 (24.6)	0.749	3 (23.1)	0.945	6 (35.3)	0.351
Peripheral	50 (61.7)	33 (57.9)	0.651	6 (46.2)	0.288	7 (41.2)	0.118
Central and peripheral	13 (16.0)	10 (17.5)	0.817	4 (30.8)	0.243	4 (23.5)	0.487
Malignant PE	28 (34.6	12 (21.1)	0.085	4 (30.8)	0.787	4 (23.5)	0.378
Size (mm)	45.7 ± 21.9	47.1 ± 28.0	0.755	55.0 ± 28.9	0.180	50.4 ± 36.9	0.486
Brain meta at diagnosis	15 (18.5)	6 (10.5)	0.198	2 (15.4)	0.782	2 (11.8)	0.729
Brain meta at diagnosis and during follow-up	23 (28.4)	15 (26.3)	0.788	4 (30.8)	0.861	6 (35.3)	0.571

Data are mean values ± standard deviations for continuous variables and number of patients with percentages in parentheses for categorical variables.

Bold numbers indicate significant *p*-values (< 0.05).

**Table 4:** Comparison of *ALK* positive tumors with *ALK* negative and pan-negative tumors.

Variable	*ALK* positive (*n* = 17)	*ALK* negative† (*n* = 121)	*p*	*ALK* negative‡ (*n* = 91)	*p*	Pan-negative (*n* = 13)	*p*
Age (years)	64.2 ± 14.0	63.1 ± 13.1	0.753	63.9 ± 12.5	0.929	71.9 ± 9.2	0.098
Gender			0.117		0.166	7 (53.8)	0.785
Male	10 (58.8)	47 (38.8)		37 (40.7)		6 (46.2)	
Female	7 (41.2)	74 (61.2)		54 (59.3)			
Clinical stage							
I	0 (0.0)	9 (7.4)	0.601	7 (7.7)	0.594	1 (7.7)	0.433
II	2 (11.8)	9 (7.4)	0.626	7 (7.7)	0.631	1 (7.7)	0.709
III	5 (29.4)	18 (14.9)	0.162	16 (17.6)	0.316	3 (23.1)	0.696
IV	10 (58.8)	85 (70.2)	0.341	61 (67.0)	0.513	8 (61.5)	0.880
T stage							
T1	3 (17.6)	16 (13.2)	0.705	13 (14.3)	0.715	0 (0.0)	0.238
T2	4 (23.5)	35 (28.9)	0.779	23 (25.3)	0.878	4 (30.8)	0.698
T3	2 (11.8)	22 (18.2)	0.957	15 (16.5)	0.907	1 (7.7)	0.613
T4	2 (11.8)	48 (39.7)	0.905	40 (44.0)	0.832	8 (61.5)	0.269
LN meta	15 (88.2)	90 (74.4)	0.361	65 (71.4)	0.228	10 (76.9)	0.628
N stage							
N0	2 (11.8)	31 (25.6)	0.361	26 (28.6)	0.228	3 (23.1)	0.628
N1	1 (5.9)	15 (12.4)	0.693	8 (8.8)	0.678	2 (15.4)	0.565
N2	9 (52.9)	30 (24.8)	**0.022**	25 (27.5)	**0.038**	3 (23.1)	0.098
N3	5 (29.4)	45 (37.2)	0.532	32 (35.2)	0.646	5 (38.5)	0.705
Extrathoracic meta	5 (29.4)	60 (49.6)	0.119	39 (42.9)	0.300	5 (38.5)	0.705
M stage							
M0	7 (41.2)	36 (29.8)	0.341	30 (33.0)	0.513	5 (38.5)	0.880
M1a	5 (29.4)	25 (20.7)	0.529	22 (24.2)	0.761	3 (23.1)	0.696
M1b	0 (0.0)	18 (14.9)	0.127	14 (15.4)	0.120	1 (7.7)	0.433
M1c	5 (29.4)	42 (34.7)	0.666	25 (27.5)	0.870	4 (30.8)	0.936
Localization							
Right upper lobe	0 (0.0)	31 (25.6)	**0.013**	24 (26.4)	**0.012**	0 (0.0)	-
Right lower lobe	2 (11.8)	12 (9.9)	0.683	11 (12.1)	0.970	3 (23.1)	0.628
Middle lobe	2 (11.8)	6 (5.0)	0.256	3 (3.3)	0.175	1 (7.7)	0.709
Left upper lobe	4 (23.5)	23 (19.0)	0.744	17 (18.7)	0.739	3 (23.1)	0.977
Left lower lobe	3 (17.6)	19 (15.7)	0.735	10 (11.0)	0.427	2 (15.4)	0.869
Lingula	1 (5.9)	1 (0.8)	0.232	1 (1.1)	0.291	0 (0.0)	0.281
Mixed	5 (29.4)	29 (24.0)	0.764	25 (27.5)	0.870	4 (30.8)	0.936
Distribution							
Central	6 (35.3)	26 (21.5)	0.225	19 (20.9)	0.217	3 (23.1)	0.691
Peripheral	7 (41.2)	76 (62.8)	0.088	59 (64.8)	0.066	6 (46.2)	0.785
Central and peripheral	4 (23.5)	19 (15.7)	0.485	13 (14.3)	0.466	4 (30.8)	0.698
Malignant PE	4 (23.5)	36 (29.8)	0.778	21 (23.1)	0.968	4 (30.8)	0.698
Size (mm)	50.4 ± 36.9	45.7 ± 22.4	0.461	46.4 ± 22.3	0.543	55.0 ± 28.9	0.714
Brain meta at diagnosis	2 (11.8)	19 (15.7)	0.663	11 (12.1)	0.970	2 (15.4)	0.773
Brain meta at diagnosis and during follow-up	6 (35.3)	32 (26.4)	0.562	21 (23.1)	0.360	4 (30.8)	0.794

Data are mean values ± standard deviations for continuous variables and number of patients with percentages in parentheses for categorical variables.

^†^Patients with *EGFR* or *KRAS* mutation and unknown *ALK* mutation status included.

^‡^Analysis restricted to patients with known *ALK* mutation status. Bold numbers indicate significant *p*-values (< 0.05).

### Correlation with outcome

**Fig 2:**
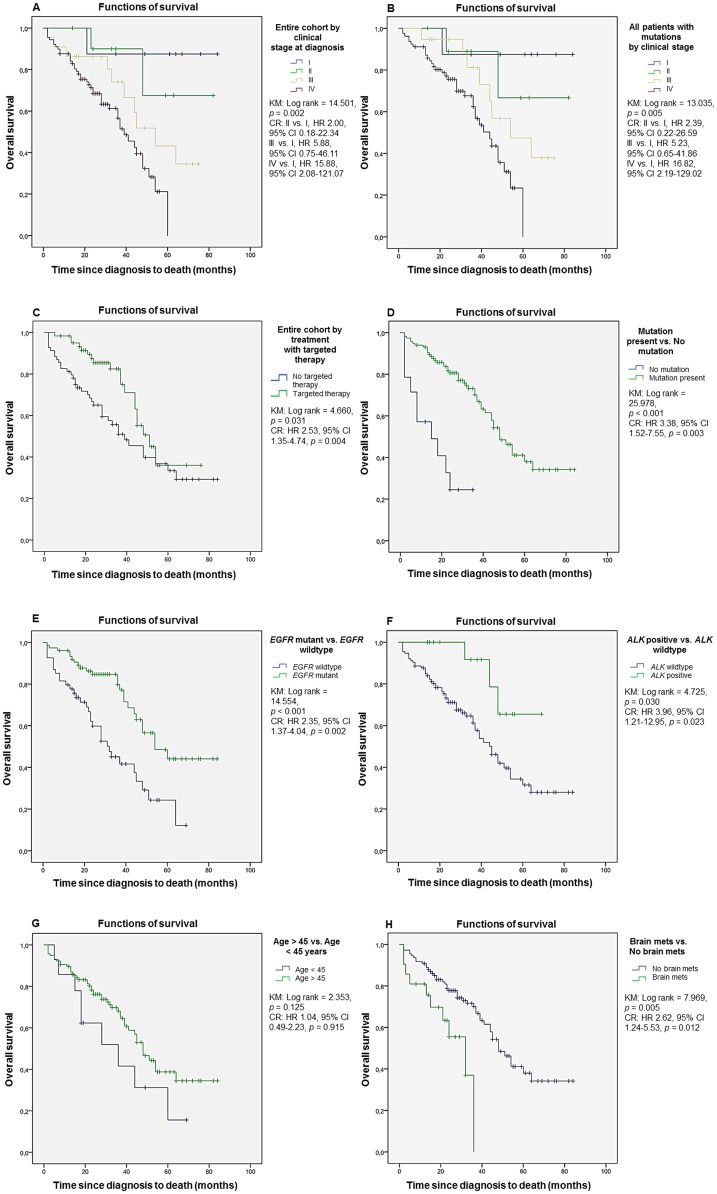
Overall survival in never smokers with lung adenocarcinoma. (A) Entire cohort by clinical stage at diagnosis. (B) All patients with mutations by clinical stage at diagnosis. (C) Entire cohort by treatment with targeted therapy. (D) All patients by presence of oncogenic driver mutations. (E) All patients by *EGFR* mutation status. (F) All patients by *ALK* rearrangement status. (G) Entire cohort by age at diagnosis. (H) All patients by presence of brain metastases at diagnosis. KM, Kaplan Meier; CR, Cox regression.

Early clinical stage (in the entire study cohort–*p* = 0.002, Fig 2a; in all patients with mutations in their tumors–*p* = 0.005, Fig 2b; in all patients with *EGFR* mutant and *ALK* rearranged tumors–*p* = 0.005) and treatment with targeted therapy (in the entire study cohort–HR 2.53, 95% CI 1.35–4.74, *p* = 0.004, Fig 2c; in all patients with mutations in their tumors–HR 2.61, 95% CI 1.39–4.90, *p* = 0.003; and in the cohort with *EGFR* mutation or *ALK* rearrangement–HR 2.22, 95% CI 0.99–4.99, *p* = 0.043) were independently associated with longer mOS. Patients with mutations in their tumors had significantly longer mOS compared to patients without mutations (mOS 52.2 vs. 16.9 mo; HR 3.38; 95% CI 1.52–7.55; *p* = 0.003, Fig 2d), and patients with *EGFR* mutated tumors had significantly better outcome compared to patients with *EGFR* wildtype and pan-negative tumors (mOS 57.8 vs. 35.0 mo, HR 2.35, 95% CI 1.37–4.04, *p* = 0.002, Fig 2e, and mOS 57.8 vs. 16.9 mo, HR 7.80, 95% CI 3.28–18.55, *p* < 0.001). Among the covariates included in the multivariable model (age, gender, clinical stage, treatment) only treatment with targeted therapy and clinical stage were found significant. In the subset of patients with *EGFR* mutant lung adenocarcinoma, no significant survival difference was found between patients with tumors carrying exon 19 deletions and patients harboring exon 21 p.L858R point mutation (mOS 47.0 vs. 58.7 mo, HR 1.02, 95% CI 0.34–3.08, *p* = 0.977). In contrast, patients with *ALK* rearranged tumors had significantly longer OS compared to the *ALK* wildtype and pan-negative cohorts (mOS 59.9 vs. 46.5 mo, HR 3.96, 95% CI 1.21–12.95, *p* = 0.023, Fig 2f and mOS 59.9 vs.16.9 mo, HR 34.78, 95% CI 3.48–34.65, *p* = 0.003). Age, gender, clinical stage and treatment with targeted therapy proved to be significant covariates in the multivariable regression model, and statistical significance for all variables was preserved when the analysis was restricted to the subset of patients for whom *ALK* mutation status was known. Patients > 45 years had insignificantly longer OS compared to patients < 45 years (mOS 50.7 vs. 36.2 mo, HR 1.04, 95% CI 0.49–2.23, *p* = 0.915, Fig 2g), while patients with brain metastases at diagnosis had significantly shorter OS compared to patients without brain metastases at diagnosis (mOS 24.6 vs. 51.5 mo, HR 2.62, 95% CI 1.24–5.53, *p* = 0.012, Fig 2h).

## Discussion

The study presents the molecular, demographic and clinicopathologic features of never smoker patients with lung adenocarcinoma, diagnosed at a single institution in Switzerland. We comprehensively analyzed associations between mutation status and clinicopathologic characteristics and outcome.

To the best of our knowledge, only two previous studies have thoroughly investigated the genomic characteristics of LCINS in Western populations [1,6]. In the first study, a large multicenter series from France, comprising 384 never smokers with NSCLC (adenocarcinoma histology– 85%), Couraud et al. [6] reported *ALK* rearrangement and *EGFR*, *KRAS*, *BRAF*, *ERBB2* and *PIK3CA* mutation in 13%, 43%, 7%, 5%, 4% and 2% of tested patients, respectively, using different genotyping methods in overall 75 participating centers. The authors did not comprehensively analyze associations between mutation status and clinicopathologic and demographic patient characteristics, instead the study focused on epidemiologic data including information on exposure to occupational carcinogens and passive smoker exposure [6]. In the second study, a large retrospective single center series from Canada, comprising 712 never smoking lung cancer patients (adenocarcinoma histology– 87%), 515 of whom had tumor tissue available for molecular analysis, Korpanty et al. [1] reported *EGFR*, *KRAS*, *TP53*, *ERBB2*, *BRAF* and *PIK3CA* mutation and *ALK* rearrangement in 52.2%, 2.3%, 1.4%, 1,0%, 0.4%, 0.4% and 7.6% of patients, respectively, using MassARRAY technology (Sequenom, San Diego, CA) or MiSeq (Illumina, San Diego, CA) NGS personal genomics platforms as testing methods. In accordance with the above-mentioned studies [1,6] and the results of investigations from Asian populations [7,18,19] *EGFR* mutation was the most commonly encountered mutation type in our study population (58.7%), and the majority (55.6%) of *EGFR* mutations were deletions in exon 19. It is well known that the reported *EGFR* mutation rates in patients with lung adenocarcinoma vary widely among different populations worldwide (ranging from 10–20% in European and North American cohorts [20–23] to more than 50% in Asian populations [24,25]), and that *EGFR* mutation status is significantly associated with female gender and never smoking status [20–25]. When we confined the analysis to female never smokers in our cohort, we achieved a high *EGFR* mutation rate of 63.0%, which is consistent with previous reports showing *EGFR* mutation rates reaching up to 60% when focusing on female never smoker patients [24–26]. *ALK* translocations, detected in 3–7% of non-selected NSCLC cohorts [27–29], are reported to occur more commonly in non-smokers, lung adenocarcinomas and non-Asian vs. Asian populations [30]. The frequency of *ALK* rearrangements in our study (12.3%) was similar to that reported for never smoker subgroups in previous investigations (range, 4.5%-16.4% [7,18,28,29,31]). In a recent study, we analyzed mutation frequencies and associations between mutational status and clinicopathologic patient characteristics in a non-selected representative cohort of Swiss patients with newly diagnosed lung adenocarcinoma the majority of whom (354/469, 75.5%) were ever smokers (current smokers or ex-smokers) [17]. Compared with the current study, we found a significantly higher percentage of *KRAS* mutation (159/469, 33.9%), which was the most common mutation in this non-selected patient cohort, and lower frequencies of *EGFR* mutation (90/469, 19.2%) and *ALK* rearrangement (28/469, 6.0%). Similar to the results of the current study, *EGFR* mutated tumors more frequently had (multiple) extrathoracic metastases at diagnosis and tended to occur more frequently in the right upper lobe compared to *EGFR* wildtype and *ALK* rearranged tumors, while *ALK* positive lung adenocarcinomas were less frequently located in the right upper lobe compared to *ALK* wildtype tumors. In both studies, we found that *ALK*-rearranged tumors were more commonly associated with ipsilateral mediastinal or subcarinal lymph node metastasis (N2) compared to *ALK* wildtype and *EGFR* mutated lung adenocarcinoma. In a non-selected stage I-III NSCLC population, Paik et al. [32] reported lower tumor stage (pT1) and significantly higher frequency of lymph node metastases in *ALK* FISH-positive NSCLC cases compared to *ALK* FISH-negative NSCLC cases. The authors suggested that *ALK*-rearranged lung cancer might have unique biological features with a tendency to early lymph node metastasis despite small primary tumor size, which could explain higher incidences of *ALK* rearrangement in advanced NSCLC when compared with surgically resectable lung cancer [32].

Recent studies have suggested that never smoker patients with tumors harboring mutations may have significantly longer mOS compared to patients without identifiable mutations [1]. Besides, never smokers with *ALK* rearranged NSCLC are reported to have significantly better outcome compared to the *ALK* wildtype and pan-negative cohorts [1,7]. In accordance with these reports, patients with oncogenic driver mutations in our study had significantly longer mOS compared to patients without identifiable mutations, and patients with *ALK* translocation had significantly better outcome compared to patients without *ALK* rearrangement and the pan-negative cohort. Similarly, the presence of *EGFR* mutation in our study was significantly associated with longer OS in univariable and multivariable analysis, while, consistent with the results reported by Korpanty et al. [1] and in contrast to previous reports [33,34], no significant survival difference was found between patients with tumors harboring *EGFR* exon 19 deletions and patients with tumors harboring *EGFR* exon 21 mutations.

The initial onset of brain metastases is generally considered an unfavorable prognostic factor that increases the risk of death [35]. In a recent study, Korpanty et al. [1] found no significant survival difference in patients with and those without brain metastases at diagnosis. The authors suggested that the lack of survival difference may be related to the high proportion (~80%) of tumors harboring *EGFR* mutation or *ALK* translocation among patients with brain metastases in their study [1], as these mutations are known to be associated with favorable response of CNS disease to targeted tyrosine kinase inhibitors and higher response rates to whole brain radiation therapy compared to wildtype lung cancer [36–40]. In our study, patients with brain metastases at diagnosis had significantly shorter mOS compared to patients without brain metastases at diagnosis despite the fact that 81.0% of patients with brain metastases at presentation had tumors with two most common targetable mutations (71.4%–*EGFR*, 9.5%–*ALK*).

NSCLC diagnosed in young patients is a rare entity, with incidences ranging between 1.3 and 5.3% among patients ≤ 45 years at diagnosis [4,41–43]. The genomics and clinical characteristics of this disease are poorly understood, and studying the relationship between age and genotype in young NSCLC patients is challenging due to multiple confounding factors such as gender, race and smoking status [44]. Previous reports have indicated that younger age may be associated with an increased likelihood of harboring oncogenic driver mutations in patients with NSCLC [44,45]. Besides, recent data suggest that *ALK* rearrangements occur more frequently in younger NSCLC patients compared to older patients with lung cancer [44–47], whereas *KRAS* mutations appear to be less frequent in the younger patient cohort [44,45]. Regarding *EGFR* mutation frequency, a study by Sacher et al. [44] comprising 2237 NSCLC patients found an increased likelihood of *EGFR* mutations in patients diagnosed with NSCLC at a younger age, while Tanaka et al. [45] reported a significantly lower frequency of *EGFR* mutations in 81 lung adenocarcinoma patients ≤ 40 years compared with 1665 lung adenocarcinoma patients > 40 years at initial diagnosis. In our study, focusing on never smoker patients with adenocarcinoma histology, we found no significant differences in the frequency of *EGFR* mutation, *KRAS* mutation or *ALK* translocation between patients < 45 years and patients ≥ 45 years. 92.9% of patients < 45 years in our study had potentially targetable genetic alterations in *EGFR*, *ALK*, *ROS1* and *MET*, 78.6% had *EGFR* mutation or *ALK* rearrangement (71.4%–*EGFR*, 7.1%–*ALK*), and 14.3% had tumors harboring *TP53* mutation. Consistent with previous results [44,45], patients < 45 years had shorter mOS compared to patients ≥ 45 years, although the difference in survival was not statistically significant in our cohort. It has been suggested that the worse prognosis in young NSCLC patients could be partly related to the significantly higher prevalence of *TP53* mutations in young patients with lung adenocarcinoma [45,48]. However, further studies are needed to investigate the underlying mechanisms that explain the more aggressive biology of lung cancer in younger patients.

There are several limitations to this study. First, this is a retrospective analysis of highly selected patients diagnosed and treated from 2011–2018 at a single tertiary referral academic institution. During that time, different sequencing methods were used, and we are aware of the heterogeneity of molecular testing methods and the fact that molecular pathology data were not completely comprehensive for all patients, which might have influenced the results of the current study. In addition, due to rapidly changing treatment guidelines different treatment regimens and sequence of these therapies were applied during the study period, including the incorporation of targeted therapy in routine practice for selected patients, and we acknowledge that our analyses are limited by the heterogeneity of treatment modalities and different combinations of therapies. Second, we did not attempt to identify comorbidities or specific exposures that may contribute to lung cancer risk including exposure to other carcinogens such as asbestos, radon, radiation therapy, and various other exposures in environmental, medical and/or occupational settings. Given the retrospective nature of the study, our analyses are further limited by uncertainties about errors and incompleteness of information about smoking exposure. Last, we did not analyze associations between programmed death-ligand 1 (PD-L1) expression and molecular features or outcome, although recent studies have shown that the predictive value of PD-L1 in NSCLC patients may be influenced by oncogenic driver mutation status [49]. Future prospective studies are needed to comprehensively investigate PD-L1 expression and the efficacy of PD-1/PD-L1 inhibitor therapy in different subsets of never smoker patients with oncogenic driver mutations.

## Conclusion

This is the first comprehensive analysis of molecular, clinicopathologic and survival data of never smokers with lung adenocarcinoma diagnosed at a tertiary referral academic hospital in Switzerland. There was a high incidence of oncogenic driver mutations in our study population, and *EGFR* mutation and *ALK* rearrangement were the most common genetic alterations. *EGFR* mutated tumors were more commonly associated with extrathoracic metastases at diagnosis compared to EGFR wildtype tumors, while *ALK* rearranged tumors more commonly had ipsilateral mediastinal or subcarinal lymph node metastasis compared to *EGFR* mutated and *ALK* wildtype tumors. Patients < 45 years more commonly showed lymph node metastasis and extrathoracic metastases compared to patients > 45 years. Consistent with previous reports never smokers with oncogenic driver mutations had significantly longer OS compared to patients without identifiable mutations. Likewise, there were significant differences in survival between patients with *EGFR* mutated vs. *EGFR* wildtype tumors, *EGFR* mutated vs. pan-negative tumors, *ALK* rearranged vs. *ALK* wildtype tumors and *ALK* rearranged vs. pan-negative tumors (Table 5).

**Table 5:** Most relevant study results.

**Most common mutations:**
*EGFR* (81/138,58.7%), *ALK* (17/138, 12.3%), *TP 53* (8/138, 5.8%), *MET* (8/138, 5.8%), *KRAS* (6/138, 4.3%), *ERBB2* (6/138, 4.3%), *PIK3CA* (4/138, 2.9%), *BRAF* (3/138, 2.2%), *ROS1* (2/138, 1.4%), *RET* (2/138, 1.4%)
**Correlation with clinicopathologic characteristics:**
*EGFR* mutated vs. *EGFR* wildtype tumors
- extrathoracic metastases at diagnosis (55.6% vs. 35.1%, *p* = 0.018)
*ALK* rearranged vs. *ALK* wildtype tumors
- ipsilateral mediastinal or subcarinal lymph node metastasis (52.9% vs. 24.8%, *p* = 0.022)
*ALK* rearranged vs. *EGFR* mutated tumors
- ipsilateral mediastinal or subcarinal lymph node metastasis (52.9% vs. 22.2%, *p* = 0.016)
Patients < 45 years vs. patients > 45 years
- lymph node metastasis (100.0% vs. 73.4%, *p* = 0.022)
- extrathoracic metastases (78.6% vs. 43.5%, *p* = 0.013)
**Correlation with outcome:**
Patients with mutations vs. patients without mutations
- mOS 52.2 vs. 16.9 mo, *p* = 0.003
Patients with *EGFR* mutated vs. *EGFR* wildtype tumors
- mOS 57.8 vs. 35.0 mo, *p* = 0.002
Patients with *EGFR* mutated vs. pan-negative tumors
- mOS 57.8 vs. 16.9 mo, *p* < 0.001
Patients with *ALK* rearranged vs. *ALK* wildtype tumors
- mOS 59.9 vs. 46.5 mo, *p* = 0.023
Patients with *ALK* rearranged vs. pan-negative tumors
- mOS 59.9 vs. 16.9 mo, *p* = 0.003
Patients with brain metastasis at diagnosis vs. patients without brain metastasis at diagnosis
- mOS 24.6 vs. 51.5 mo, *p* = 0.012

mOS: median overall survival.

## Supporting information

S1 Table Detailed characteristics of multiple mutations/translocations. (DOCX)

S2 Table Comparison of male and female never smokers. (DOCX)

S3 Table Comparison of mutated and pan-negative tumors. (DOCX)

S4 Table Comparison of EGFR exon 19 deletions and L858R mutations. (DOCX)

S5 Table Comparison of patients with and without brain metastases at diagnosis. (DOCX)

S6 Table Comparison of patients with and without brain metastases at diagnosis and during follow-up. (DOCX)

S7 Table Comparison of patients > 45 years and patients < 45 years. (DOCX)

## Abbreviations

*ALK*: Anaplastic lymphoma kinase
*BRAF*: B rapidly accelerated fibrosarcoma
*EGFR*: Epidermal growth factor receptor
*ERBB2*: Erb-b2 tyrosine kinase 2
FISH: Fluorescence in situ hybridization
ICC: Immunocytochemistry
IHC: Immunohistochemistry
*KRAS*: Kirsten rat sarcoma
LCINS: Lung cancer in never smokers
*MET*: Mesenchymal epithelial transition proto-oncogene
NGS: Next-generation sequencing
NSCLC: Non-small cell lung cancer
OS: Overall survival
PCR: polymerase chain reaction
PD-L1: Programmed death-ligand 1
*PIK3CA*: Phosphatidylinositol-3 kinase catalytic subunit alpha
*RET*: Rearranged during transfection proto-oncogene
*ROS1*: ROS proto-oncogene 1
SS: Sanger sequencing
*TP53*: Tumor suppressor protein 53
