# Supplementary material for: Oncogenic driver mutations in Swiss never smoker patients with lung adenocarcinoma and correlation with clinicopathologic characteristics and outcome
Source: PLoS One. 2019 Aug 6;14(8):e0220691. doi: 10.1371/journal.pone.0220691 (PMC6684066; doi:10.1371/journal.pone.0220691)
Supplement: S1 Table — (DOCX) [file pone.0220691.s001.docx]

**S1 Table. Detailed characteristics of multiple mutations/translocations.**

| Multiple mutations (*n* = 21) | | |
| --- | --- | --- |
| Mutation 1 | Mutation(s)/Translocation(s) 2 (3) |  |
| *EGFR* p.L858R | *EGFR* p.E709G | *EGFR* double mutations (*n* = 9) |
| *EGFR* p.L858R | *EGFR* p.E709A |  |
| *EGFR* p.L858R | *EGFR* p.K860I |  |
| *EGFR* p.L858R | *EGFR* p.D761Y |  |
| *EGFR* p.G719A | *EGFR* p.S768I |  |
| *EGFR* p.G719A | *EGFR* p.L833V |  |
| *EGFR* p.G719C | *EGFR* p.S768I |  |
| *EGFR* p.T854A | *EGFR* p.V774M |  |
| *EGFR* p.E746_S752delinsV | *EGFR* p.C797S; *PIK3CA* p.E545K | *EGFR* and other genes (*n* =9) |
| *EGFR* p.L858R | *TP53* p.V197E |  |
| *EGFR* p.L747_P753delinsS | *PIK3CA* p.E545K |  |
| *EGFR* p.L858R | *TP53* R249T |  |
| *EGFR* p.S752_I759del | *TP53* p.C238R |  |
| *EGFR* p.L858R | *TP53* p.P278S |  |
| *EGFR* p.L858R | *PIK3CA* p.E542K; *TP53* p.H179P |  |
| *EGFR* p.E746_A750del | *ERBB2* p.E770_A771insAYVM |  |
| *EGFR* p.H773_V774insAH | *MET* p.T1010I |  |
| *KRAS* p.G12C | *PIK3CG* p.P311R | *KRAS* and other genes (*n* = 1) |
| *MET* p.E168D | *CTNNB1* p.T42S; PARP1 p.K352R |  |
| *MET* p.R988C | *TP53* p.W146* |  |
| *TP53* p.Y234C | *TET1* p.V128F |  |
