# Supplementary material for: Oncogenic driver mutations in Swiss never smoker patients with lung adenocarcinoma and correlation with clinicopathologic characteristics and outcome
Source: PLoS One. 2019 Aug 6;14(8):e0220691. doi: 10.1371/journal.pone.0220691 (PMC6684066; doi:10.1371/journal.pone.0220691)
Supplement: S2 Table — (DOCX) [file pone.0220691.s002.docx]

**S2 Table. Comparison of male and female never smokers.**

| Variable | men (*n* = 57) | women (*n* = 81) | *p* |
| --- | --- | --- | --- |
| Age (years) | 62.1 ± 13.3 | 64.1 ± 13.1 | 0.367 |
| Clinical stage |  |  |  |
| I | 5 (8.8) | 4 (4.9) | 0.488 |
| II | 3 (5.3) | 8 (9.9) | 0.525 |
| III | 9 (15.8) | 14 (17.3) | 0.817 |
| IV | 40 (70.2) | 55 (67.9) | 0.776 |
| T stage |  |  |  |
| T1 | 11 (19.3) | 8 (9.9) | 0.114 |
| T2 | 14 (24.6) | 25 (30.9) | 0.418 |
| T3 | 9 (15.8) | 16 (19.8) | 0.552 |
| T4 | 23 (40.4) | 32 (39.5) | 0.921 |
| LN metastasis/-es | 43 (75.4) | 62 (76.5) | 0.881 |
| N stage |  |  |  |
| N0 | 14 (24.6) | 19 (23.5) | 0.881 |
| N1 | 4 (7.0) | 12 (14.8) | 0.159 |
| N2 | 16 (28.1) | 23 (28.4) | 0.967 |
| N3 | 23 (40.4) | 27 (33.3) | 0.398 |
| Extrathoracic metastasis/-es | 29 (50.9) | 36 (44.4) | 0.456 |
| M stage |  |  |  |
| M0 | 17 (29.8) | 26 (32.1) | 0.776 |
| M1a | 11 (19.3) | 19 (23.5) | 0.560 |
| M1b | 9 (15.8) | 9 (11.1) | 0.422 |
| M1c | 20 (35.1) | 27 (33.3) | 0.830 |
| Brain metastases at diagnosis | 8 (14.0) | 13 (16.0) | 0.746 |
| Brain metastases at diagnosis | 17 (29.8) | 21 (25.9) | 0.614 |
| and during follow-up |  |  |  |
| Localization |  |  |  |
| Right upper lobe | 10 (17.5) | 21 (25.9) | 0.245 |
| Right lower lobe | 6 (10.5) | 8 (9.9) | 0.901 |
| Middle lobe | 4 (7.0) | 4 (4.9) | 0.717 |
| Left upper lobe | 12 (21.1) | 15 (18.5) | 0.712 |
| Left lower lobe | 10 (17.5) | 12 (14.8) | 0.666 |
| Lingula | 0 (0.0) | 2 (2.5) | 0.512 |
| Involvement of two lobes | 15 (26.3) | 19 (23.5) | 0.701 |
| Distribution |  |  |  |
| Central | 15 (26.3) | 17 (21.0) | 0.465 |
| Peripheral | 35 (61.4) | 48 (59.3) | 0.800 |
| Central and peripheral | 7 (12.3) | 16 (19.8) | 0.246 |
| Malignant pleural effusion | 18 (31.6) |  |  |
| Size (mm) | 45.4 ± 25.4 | 46.9 ± 24.0 | 0.718 |
| *EGFR* | 30 (52.6) | 51 (63.0) | 0.225 |
| *ALK* | 10 (17.5) | 7 (8.6) | 0.117 |
| *KRAS* | 4 (7.0) | 3 (3.7) | 0.447 |
| *BRAF* | 0 (0.0) | 3 (3.7) | 0.267 |
| *RET* | 1 (1.8) | 1 (1.2) | 0.803 |
| *ROS1* | 0 (0.0) | 4 (4.9) | 0.142 |
| *PIK3CA* | 2 (3.5) | 2 (2.5) | 0.722 |
| *ERBB2* | 2 (3.5) | 4 (4.9) | 0.682 |
| *MET* | 2 (3.5) | 6 (7.4) | 0.470 |
| Other | 5 (8.8) | 6 (7.4) | 0.761 |

Data are mean values ± standard deviations for continuous variables and number of patients with percentages in parentheses for categorical variables.
