# Supplementary material for: Oncogenic driver mutations in Swiss never smoker patients with lung adenocarcinoma and correlation with clinicopathologic characteristics and outcome
Source: PLoS One. 2019 Aug 6;14(8):e0220691. doi: 10.1371/journal.pone.0220691 (PMC6684066; doi:10.1371/journal.pone.0220691)
Supplement: S3 Table — (DOCX) [file pone.0220691.s003.docx]

**S3 Table. Comparison of mutated and pan-negative tumors.**

| Variable | Mut. present (n = 125) | Pan-negative (n = 13) | *p* |
| --- | --- | --- | --- |
| Age (years) | 62.4 ± 13.2 | 71.9 ± 9.2 | **0.012** |
| Gender |  |  | 0.335 |
| Male | 50 (40.0) | 7 (53.8) |  |
| Female | 75 (60.0) | 6 (46.2) |  |
| Clinical stage |  |  |  |
| I | 8 (6.4) | 1 (7.7) | 0.861 |
| II | 10 (8.0) | 1 (7.7) | 0.969 |
| III | 20 (16.0) | 3 (23.1) | 0.455 |
| IV | 87 (69.5) | 8 (61.5) | 0.543 |
| T stage |  |  |  |
| T1 | 19 (15.2) | 0 (0.0) | 0.214 |
| T2 | 35 (28.0) | 4 (30.8) | 0.834 |
| T3 | 24 (19.2) | 1 (7.7) | 0.462 |
| T4 | 47 (37.6) | 8 (61.5) | 0.093 |
| LN metastasis/-es | 95 (76.0) | 10 (76.9) | 0.941 |
| N stage |  |  |  |
| N0 | 30 (24.0) | 3 (23.1) | 0.941 |
| N1 | 14 (11.2) | 2 (15.4) | 0.648 |
| N2 | 36 (18.8) | 3 (23.1) | 0.657 |
| N3 | 45 (36.0) | 5 (38.5) | 0.861 |
| Extrathoracic metastasis/-es | 60 (48.0) | 5 (38.5) | 0.512 |
| M stage |  |  |  |
| M0 | 38 (30.4) | 5 (38.5) | 0.543 |
| M1a | 27 (21.6) | 3 (23.1) | 0.903 |
| M1b | 17 (13.6) | 1 (7.7) | 0.522 |
| M1c | 43 (34.4) | 4 (30.8) | 0.791 |
| Localization |  |  |  |
| Right upper lobe | 31 (24.8) | 0 (0.0) | **0.040** |
| Right lower lobe | 11 (8.8) | 3 (23.1) | 0.129 |
| Middle lobe | 7 (5.6) | 1 (7.7) | 0.557 |
| Left upper lobe | 24 (19.2) | 3 (23.1) | 0.719 |
| Left lower lobe | 20 (16.0) | 2 (15.4) | 0.954 |
| Lingula | 2 (1.6) | 0 (0.0) | 0.528 |
| Involvement of two lobes | 30 (24.0) | 4 (30.8) | 0.735 |
| Distribution |  |  |  |
| Central | 29 (23.2) | 3 (23.1) | 0.992 |
| Peripheral | 77 (61.6) | 6 (46.2) | 0.279 |
| Central and peripheral | 19 (15.2) | 4 (30.8) | 0.231 |
| Malignant pleural effusion | 36 (28.8) | 4 (30.8) | 0.882 |
| Size (mm) | 45.4 ± 24.0 | 55.0 ± 28.9 | 0.179 |
| Brain metastases at diagnosis | 19 (15.2) | 2 (15.4) | 0.986 |
| Brain metastases at diagnosis | 34 (27.2) | 4 (30.8) | 0.753 |
| and during follow-up |  |  |  |

Data are mean values ± standard deviations for continuous variables and number of patients with percentages in parentheses for categorical variables. Bold numbers indicate significant *p*-values (< 0.05).
