# Supplementary material for: Oncogenic driver mutations in Swiss never smoker patients with lung adenocarcinoma and correlation with clinicopathologic characteristics and outcome
Source: PLoS One. 2019 Aug 6;14(8):e0220691. doi: 10.1371/journal.pone.0220691 (PMC6684066; doi:10.1371/journal.pone.0220691)
Supplement: S4 Table — (DOCX) [file pone.0220691.s004.docx]

**S4 Table. Comparison of *EGFR* exon 19 deletions and L858R mutations.**

| Variable | Exon 19 del (*n* = 45) | L858R (*n* = 26) | *p* |
| --- | --- | --- | --- |
| Age (years) | 59.0 ± 14.0 | 66.2 ± 12.8 | **0.036** |
| Gender |  |  | 0.653 |
| Male | 18 (40.0) | 9 (34.6) |  |
| Female | 27 (60.0) | 17 (65.4) |  |
| Clinical stage |  |  |  |
| I | 0 (0.0) | 3 (11.5) | 0.045 |
| II | 3 (6.7) | 1 (3.8) | 0.610 |
| III | 4 (8.9) | 4 (15.4) | 0.453 |
| IV | 38 (84.4) | 18 (69.2) | 0.130 |
| T stage |  |  |  |
| T1 | 5 (11.1) | 4 (15.4) | 0.716 |
| T2 | 17 (37.8) | 5 (19.2) | 0.103 |
| T3 | 8 (17.8) | 7 (26.9) | 0.363 |
| T4 | 15 (33.3) | 10 (38.5) | 0.663 |
| LN metastasis/-es | 35 (77.8) | 20 (76.9) | 0.934 |
| N stage |  |  |  |
| N0 | 10 (22.2) | 6 (23.1) | 0.934 |
| N1 | 6 (13.3) | 2 (7.7) | 0.701 |
| N2 | 6 (13.3) | 11 (42.3) | **0.006** |
| N3 | 23 (51.1) | 7 (26.9) | 0.047 |
| Extrathoracic metastasis/-es | 26 (57.8) | 16 (61.5) | 0.756 |
| M stage |  |  |  |
| M0 | 7 (15.6) | 8 (30.8) | 0.130 |
| M1a | 12 (26.7) | 2 (7.7) | 0.053 |
| M1b | 6 (13.3) | 6 (23.1) | 0.335 |
| M1c | 20 (44.4) | 10 (38.5) | 0.623 |
| Localization |  |  |  |
| Right upper lobe | 13 (28.9) | 6 (23.1) | 0.594 |
| Right lower lobe | 3 (6.7) | 2 (7.7) | 0.871 |
| Middle lobe | 3 (6.7) | 1 (3.8) | 0.610 |
| Left upper lobe | 7 (15.6) | 8 (30.8) | 0.130 |
| Left lower lobe | 7 (15.6) | 3 (11.5) | 0.736 |
| Lingula | 0 (0.0) | 0 (0.0) | - |
| Involvement of two lobes | 12 (26.7) | 6 (23.1) | 0.738 |
| Distribution |  |  |  |
| Central | 7 (15.6) | 10 (38.5) | **0.029** |
| Peripheral | 33 (73.3) | 10 (38.5) | **0.004** |
| Central and peripheral | 5 (11.1) | 6 (23.1) | 0.194 |
| Malignant pleural effusion | 20 (44.4) | 4 (15.4) | **0.013** |
| Size (mm) | 45.7 ± 23.3 | 45.6 ± 20.7 | 0.989 |
| Brain metastases at diagnosis | 8 (17.8) | 6 (23.1) | 0.589 |
| Brain metastases at diagnosis | 11 (24.4) | 9 (34.6) | 0.359 |
| and during follow-up |  |  |  |

Data are mean values ± standard deviations for continuous variables and number of patients with percentages in parentheses for categorical variables. Bold numbers indicate significant *p*-values (< 0.05).
