# Supplementary material for: Oncogenic driver mutations in Swiss never smoker patients with lung adenocarcinoma and correlation with clinicopathologic characteristics and outcome
Source: PLoS One. 2019 Aug 6;14(8):e0220691. doi: 10.1371/journal.pone.0220691 (PMC6684066; doi:10.1371/journal.pone.0220691)
Supplement: S5 Table — (DOCX) [file pone.0220691.s005.docx]

**S5 Table. Comparison of patients with and without brain metastases at diagnosis.**

| Variable | Patients with BM | Patients without BM | *p* |
| --- | --- | --- | --- |
|  | at diagnosis (*n* = 21) | at diagnosis (*n* = 117) |  |
| Age (years) | 58.3 ± 14.3 | 64.2 ± 12.8 | 0.059 |
| Gender |  |  | 0.746 |
| Male | 8 (38.1) | 49 (41.9) |  |
| Female | 13 (61.9) | 68 (58.1) |  |
| T stage |  |  |  |
| T1 | 1 (4.8) | 18 (15.4) | 0.306 |
| T2 | 6 (28.6) | 33 (28.2) | 0.973 |
| T3 | 1 (4.8) | 24 (20.5) | 0.123 |
| T4 | 13 (61.9) | 42 (35.9) | **0.025** |
| LN metastasis/-es | 19 (90.5) | 86 (73.5) | 0.093 |
| N stage |  |  |  |
| N0 | 2 (9.5) | 31 (26.5) | 0.093 |
| N1 | 0 (0.0) | 16 (13.7) | 0.131 |
| N2 | 6 (28.6) | 33 (28.2) | 0.973 |
| N3 | 13 (61.9) | 37 (31.6) | **0.008** |
| Localization |  |  |  |
| Right upper lobe | 5 (23.8) | 26 (22.2) | 0.873 |
| Right lower lobe | 2 (9.5) | 12 (10.3) | 0.918 |
| Middle lobe | 1 (4.8) | 7 (6.0) | 0.821 |
| Left upper lobe | 3 (14.3) | 24 (20.5) | 0.765 |
| Left lower lobe | 6 (28.6) | 16 (13.7) | 0.106 |
| Lingula | 0 (0.0) | 2 (1.7) | 0.415 |
| Involvement of two lobes | 4 (19.0) | 30 (25.6) | 0.519 |
| Distribution |  |  |  |
| Central | 6 (28.6) | 26 (22.2) | 0.576 |
| Peripheral | 11 (52.4) | 72 (61.5) | 0.430 |
| Central and peripheral | 4 (19.0) | 19 (16.2) | 0.753 |
| Malignant pleural effusion | 7 (33.3) | 33 (28.2) | 0.633 |
| Size (mm) | 44.7 ± 17.7 | 46.6 ± 25.6 | 0.743 |
| *EGFR* | 15 (71.4) | 66 (56.4) | 0.198 |
| *ALK* | 2 (9.5) | 15 (12.8) | 0.663 |
| *KRAS* | 1 (4.8) | 6 (5.1) | 0.943 |
| *BRAF* | 0 (0.0) | 3 (2.6) | 0.317 |
| *RET* | 0 (0.0) | 2 (1.7) | 0.415 |
| *ROS1* | 0 (0.0) | 4 (3.4) | 0.247 |
| *PIK3CA* | 1 (4.8) | 3 (2.6) | 0.487 |
| *ERBB2* | 0 (0.0) | 6 (5.1) | 0.590 |
| *MET* | 1 (4.8) | 7 (6.0) | 0.821 |
| Other | 1 (4.8) | 10 (8.4) | 0.531 |

Data are mean values ± standard deviations for continuous variables and number of patients with percentages in parentheses for categorical variables. BM, brain metastases. Bold numbers indicate significant *p*-values (< 0.05).
