# Supplementary material for: Oncogenic driver mutations in Swiss never smoker patients with lung adenocarcinoma and correlation with clinicopathologic characteristics and outcome
Source: PLoS One. 2019 Aug 6;14(8):e0220691. doi: 10.1371/journal.pone.0220691 (PMC6684066; doi:10.1371/journal.pone.0220691)
Supplement: S6 Table — (DOCX) [file pone.0220691.s006.docx]

**S6 Table. Comparison of patients with and without brain metastases at diagnosis and during follow-up.**

| Variable | Patients with BM | Patients without BM | *p* |
| --- | --- | --- | --- |
|  | at diagnosis and during | at diagnosis and during |  |
|  | follow-up (*n* = 38) | follow-up (*n* = 100) |  |
| Age (years) | 59.0 ± 14.0 | 64.9 ± 12.5 | **0.017** |
| Gender |  |  | 0.614 |
| Male | 17 (44.7) | 40 (40.0) |  |
| Female | 21 (55.3) | 60 (60.0) |  |
| T stage |  |  |  |
| T1 | 4 (10.5) | 15 (15.0) | 0.496 |
| T2 | 12 (31.6) | 27 (27.0) | 0.594 |
| T3 | 5 (13.2) | 20 (20.0) | 0.351 |
| T4 | 17 (44.7) | 38 (38.0) | 0.470 |
| LN metastasis/-es | 35 (92.1) | 70 (70.0) | **0.007** |
| N stage |  |  |  |
| N0 | 3 (7.9) | 30 (30.0) | **0.007** |
| N1 | 4 (10.5) | 12 (12.0) | 0.807 |
| N2 | 14 (36.8) | 25 (25.0) | 0.168 |
| N3 | 17 (44.7) | 33 (33.0) | 0.200 |
| Localization |  |  |  |
| Right upper lobe | 7 (18.4) | 24 (24.0) | 0.483 |
| Right lower lobe | 7 (18.4) | 7 (7.0) | 0.061 |
| Middle lobe | 4 (10.5) | 4 (4.0) | 0.215 |
| Left upper lobe | 4 (10.5) | 23 (23.0) | 0.099 |
| Left lower lobe | 9 (23.7) | 13 (13.0) | 0.126 |
| Lingula | 1 (2.6) | 1 (1.0) | 0.476 |
| Involvement of two lobes | 6 (15.8) | 28 (28.0) | 0.137 |
| Distribution |  |  |  |
| Central | 11 (28.9) | 21 (21.0) | 0.323 |
| Peripheral | 21 (55.3) | 62 (62.0) | 0.470 |
| Central and peripheral | 6 (15.8) | 17 (17.0) | 0.865 |
| Malignant pleural effusion | 9 (23.7) | 31 (31.0) | 0.397 |
| Size (mm) | 42.6 ± 16.9 | 47.7 ± 26.8 | 0.191 |
| *EGFR* | 23 (60.5) | 58 (58.0) | 0.788 |
| *ALK* | 6 (15.8) | 11 (11.0) | 0.562 |
| *KRAS* | 1 (2.6) | 6 (6.0) | 0.673 |
| *BRAF* | 1 (2.6) | 2 (2.0) | 0.824 |
| *RET* | 0 (0.0) | 2 (2.0) | 0.254 |
| *ROS1* | 0 (0.0) | 4 (4.0) | 0.575 |
| *PIK3CA* | 1 (2.6) | 3 (3.0) | 0.907 |
| *ERBB2* | 1 (2.6) | 5 (5.0) | 0.522 |
| *MET* | 3 (7.9) | 5 (5.0) | 0.684 |
| Other | 2 (5.3) | 9 (9.0) | 0.727 |

Data are mean values ± standard deviations for continuous variables and number of patients with percentages in parentheses for categorical variables. BM, brain metastases. Bold numbers indicate significant *p*-values (< 0.05).
