# Supplementary material for: Oncogenic driver mutations in Swiss never smoker patients with lung adenocarcinoma and correlation with clinicopathologic characteristics and outcome
Source: PLoS One. 2019 Aug 6;14(8):e0220691. doi: 10.1371/journal.pone.0220691 (PMC6684066; doi:10.1371/journal.pone.0220691)
Supplement: S7 Table — (DOCX) [file pone.0220691.s007.docx]

**S7 Table. Comparison of patients > 45 years and patients < 45 years.**

| Variable | Age > 45 (*n* = 124) | Age < 45 (*n* = 14) | *p* |
| --- | --- | --- | --- |
| Gender |  |  | 0.901 |
| Male | 51 (41.1) | 6 (42.9) |  |
| Female | 73 (58.9) | 8 (57.1) |  |
| Clinical stage |  |  |  |
| I | 9 (7.3) | 0 (0.0) | 0.598 |
| II | 11 (8.9) | 0 (0.0) | 0.603 |
| III | 22 (17.7) | 1 (7.1) | 0.465 |
| IV | 82 (66.1) | 13 (92.9) | 0.064 |
| T stage |  |  |  |
| T1 | 16 (12.9) | 3 (21.4) | 0.410 |
| T2 | 38 (30.6) | 1 (7.1) | 0.113 |
| T3 | 23 (18.5) | 2 (14.3) | 0.687 |
| T4 | 47 (37.9) | 8 (57.1) | 0.163 |
| LN metastasis/-es | 91 (73.4) | 14 (100.0) | **0.022** |
| N stage |  |  |  |
| N0 | 33 (26.6) | 0 (0.0) | **0.022** |
| N1 | 15 (12.1) | 1 (7.1) | 0.561 |
| N2 | 35 (28.2) | 4 (28.6) | 0.978 |
| N3 | 41 (33.1) | 9 (64.3) | **0.021** |
| Extrathoracic metastasis/-es | 54 (43.5) | 11 (78.6) | **0.013** |
| M stage |  |  |  |
| M0 | 42 (33.9) | 1 (7.1) | 0.064 |
| M1a | 28 (22.6) | 2 (14.3) | 0.734 |
| M1b | 16 (12.9) | 2 (14.3) | 0.886 |
| M1c | 38 (30.6) | 9 (64.3) | **0.017** |
| Brain metastases at diagnosis | 17 (13.7) | 4 (28.6) | 0.229 |
| Brain metastases at diagnosis | 32 (25.8) | 6 (42.9) | 0.209 |
| and during follow-up |  |  |  |
| Localization |  |  |  |
| Right upper lobe | 27 (21.8) | 4 (28.6) | 0.517 |
| Right lower lobe | 12 (9.7) | 2 (14.3) | 0.635 |
| Middle lobe | 6 (4.8) | 2 (14.3) | 0.188 |
| Left upper lobe | 25 (20.2) | 2 (14.3) | 0.587 |
| Left lower lobe | 22 (17.7) | 0 (0.0) | 0.126 |
| Lingula | 2 (1.6) | 0 (0.0) | 0.511 |
| Involvement of two lobes | 30 (24.2) | 4 (28.6) | 0.747 |
| Distribution |  |  |  |
| Central | 28 (22.6) | 4 (28.6) | 0.738 |
| Peripheral  Peripheral | 76 (61.3) | 7 (50.0) | 0.413 |
| Central and peripheral | 20 (16.1) | 3 (21.4) | 0.704 |
| Malignant pleural effusion | 33 (26.6) | 7 (50.0) | 0.115 |
| Size (mm) | 46.2 ± 25.3 | 47.4 ± 16.8 | 0.864 |
| *EGFR* | 71 (57.3) | 10 (71.4) | 0.307 |
| *ALK* | 16 (12.9) | 1 (7.1) | 0.507 |
| *KRAS* | 7 (5.6) | 0 (0.0) | 0.215 |
| *BRAF* | 3 (2.4) | 0 (0.0) | 0.420 |
| *RET* | 2 (1.6) | 0 (0.0) | 0.511 |
| *ROS1* | 3 (2.4) | 1 (7.1) | 0.351 |
| *PIK3CA* | 4 (3.2) | 0 (0.0) | 0.351 |
| *ERBB2* | 6 (4.8) | 0 (0.0) | 0.252 |
| *MET* | 7 (5.6) | 1 (7.1) | 0.585 |
| Other | 9 (7.3) | 2 (14.3) | 0.309 |
|  |  |  |  |

Data are mean values ± standard deviations for continuous variables and number of patients with percentages in parentheses for categorical variables. Bold numbers indicate significant *p*-values (< 0.05).
